# What Caused an Acute Pontine Stroke in a Young Male Patient With a Patent Foramen Ovale?

**DOI:** 10.7759/cureus.91901

**Published:** 2025-09-09

**Authors:** Arpankumar Patel, Umabalan Thirupathy, Hanad Bashir, Rutikbhai Desai, Puvi Seshiah

**Affiliations:** 1 Internal Medicine, The Christ Hospital, Cincinnati, USA; 2 Internal Medicine, Saint Vincent Hospital, Worcester, USA; 3 Cardiovascular Medicine, The Christ Hospital, Cincinnati, USA; 4 Internal Medicine, Gujarat Medical &amp; Education Research Society (GMERS) Medical College &amp; Hospital, Ahmedabad, IND

**Keywords:** anticoagulation, bubble echocardiography, computed tomography, echocardiography, stroke

## Abstract

Cerebral autosomal dominant arteriopathy with subcortical infarcts and leukoencephalopathy (CADASIL) is an important cause of stroke and cognitive decline in young adults due to a mutation in the NOTCH3 gene on chromosome 19 and is often underdiagnosed.

We present to you a case of a middle-aged man who presents with acute stroke symptoms. He had a family history of recurrent transient ischemic attacks (TIAs) in his father and sister. Computed tomography (CT) of the head was negative, and magnetic resonance imaging (MRI) of the brain revealed an acute infarct involving the right pons and chronic small vessel ischemic changes. An echocardiogram showed a patent foramen ovale (PFO). It was then revealed that his sister had a questionable diagnosis of CADASIL disease. The patient’s genetic testing revealed a positive monoallelic mutation of the NOTCH3 gene, confirming the diagnosis of CADASIL disease.

CADASIL is a genetic disease that leads to acute stroke and dementia at a very young age and can often go undiagnosed in clinical practice. Recent advancements in molecular genetics and early recognition of this condition with genetic testing might help in risk stratification and preventing adverse cerebrovascular outcomes.

## Introduction

Cerebral autosomal dominant arteriopathy with subcortical infarcts and leukoencephalopathy (CADASIL) is an important cause of stroke and cognitive decline in young adults due to a mutation in the NOTCH3 gene on chromosome 19p13, which encodes a receptor expressed by vascular smooth muscle cells and is often underdiagnosed [1,2]. This results in fibrosis of small penetrating vessels, causing chronic white matter ischemia. Recurrent ischemic events may eventually lead to severe disability with dementia. It is a rare disorder with a prevalence of 4.6 per 100,000 cases as per regional analyses [3]. We present a case of a young male patient whose initial stroke-like presentation was confounded by an incidentally found patent foramen ovale (PFO) and his eventual diagnosis of CADASIL.

## Case presentation

**Figure 1:**
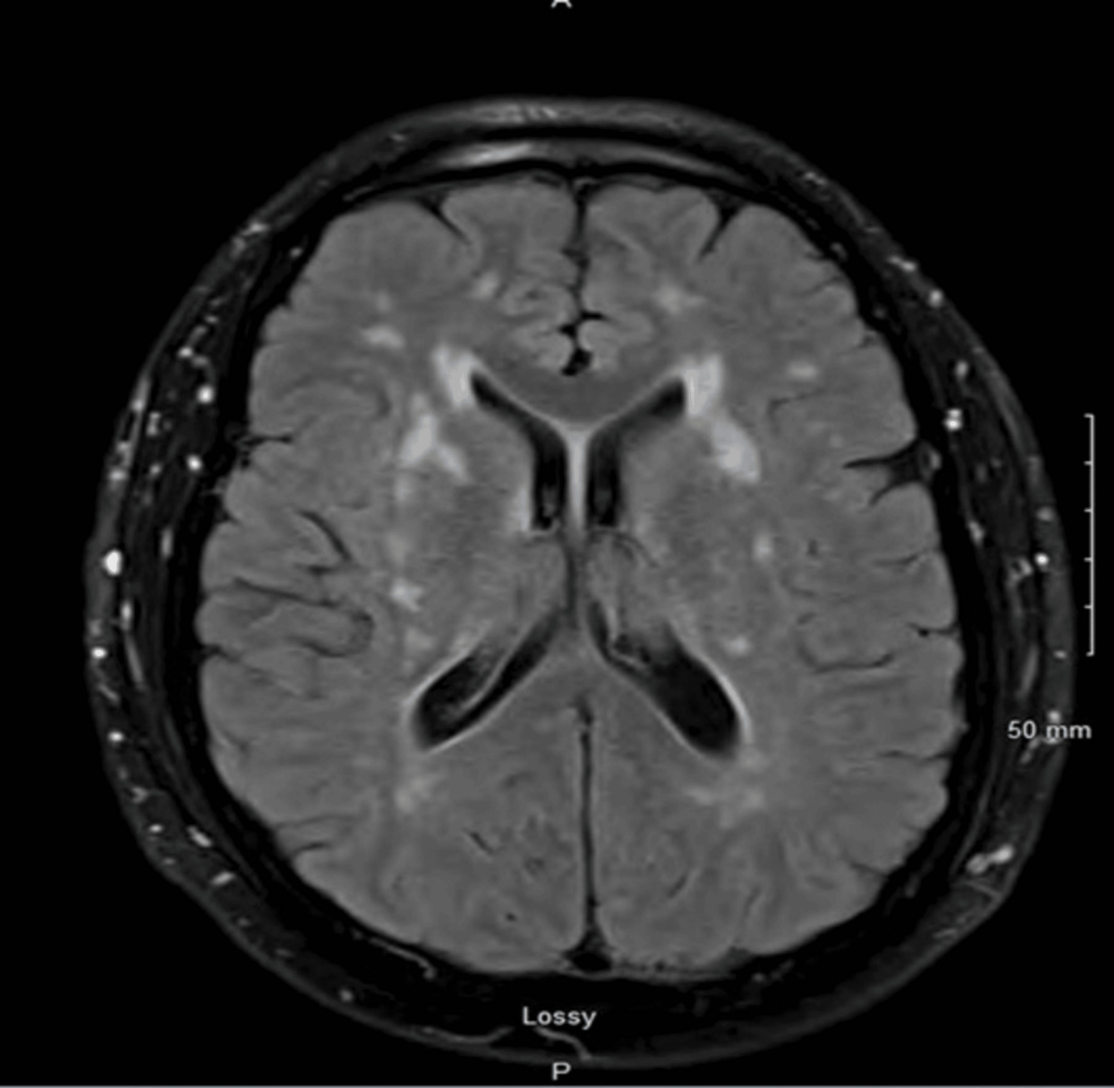
MRI images showing multiple prior infarcts, especially around the basal ganglia

**Figure 2:**
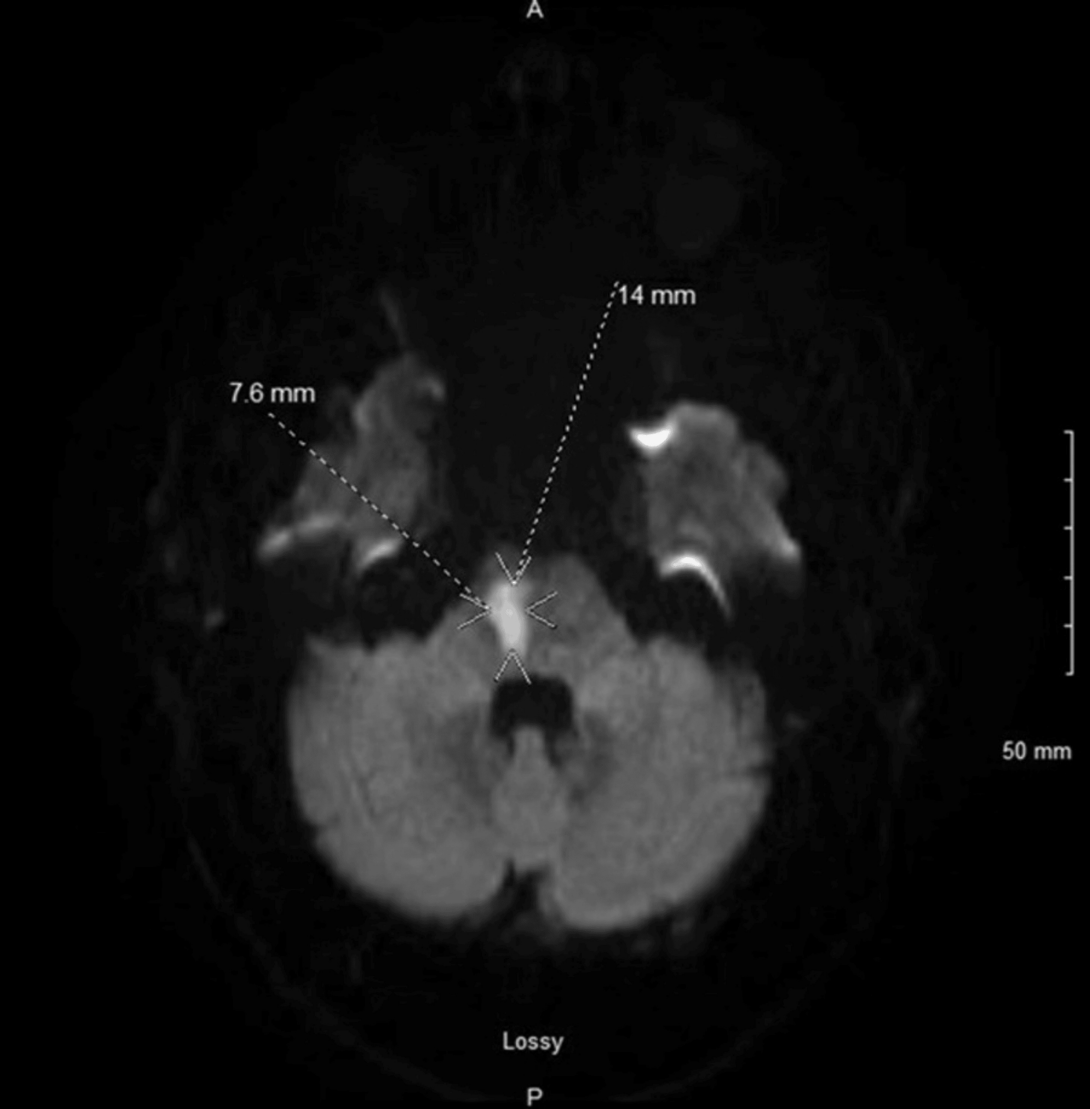
A pontine stroke of 14 mm x 7.6 mm seen on the MRI

A young, healthy man in his mid-40s with no significant past medical history presented with complaints of left-sided weakness, left facial numbness, and headache, which progressed over three days. His father and sister had a history of recurrent transient ischemic attacks (TIAs). A Computed tomography (CT) of the head was negative; however, a magnetic resonance imaging (MRI)of the brain revealed an acute infarct involving the right pons (Figure 1) and chronic small vessel ischemic changes (Figure 2).

**Figure 3:**
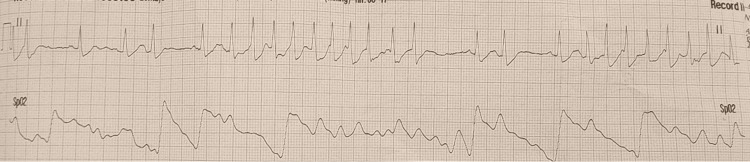
Telemetry strip showing non-sustained ventricular tachycardia

The patient's telemetry was significant for bouts of nonsustained ventricular tachycardia (Figure 3). As part of the workup to elicit the cardiogenic source of his stroke, an echocardiography was obtained, and it revealed a PFO (Video 1). Further evaluation with a venous duplex scan of the upper and lower extremities was negative for any venous clots. Since the patient had a family history of recurrent TIAs, he was discharged on aspirin, clopidogrel, and atorvastatin.

**Video 1:** Mid-esophageal short axis view of the aortic valve on the echocardiogram

In his subsequent follow-up office visit, a hypercoagulable workup and genetic study were sent to evaluate the cause of the stroke further, as the location was not consistent with a cardiogenic source, following the revelation that the patient's sister had a questionable diagnosis of CADASIL disease. His lipid profile showed elevated lipoprotein A, and he was started on icosapent ethyl; aspirin was discontinued, and apixaban was started. Hypercoagulable workup was negative. He was scheduled for PFO closure and was placed on a loop recorder. Genetic testing revealed a positive monoallelic mutation of the NOTCH3 gene, confirming the diagnosis of CADASIL disease. He was also enrolled in a CADASIL study.

## Discussion

CADASIL is an important cause of stroke caused by a mutation in the NOTCH3 gene located on chromosome 19 [1]. It can lead to cognitive impairment at an early age [2]. The prevalence of CADASIL in the community is largely unknown. Regional analyses have estimated phenotypic prevalence at around 4.6 per 100,000 cases [3]. The NOTCH3 gene, which codes for large transmembrane receptors, is usually found on chromosome 19p13.2-13.1 [4]. Transgenic mouse models suggest that extracellular NOTCH3 accumulation leads to the dysfunction of astrocytes and impaired blood-brain barrier function [5]. Due to leptomeningeal involvement, small brain arteries are involved, and autopsy findings showed lacunar infarct mostly in areas like the basal ganglia, thalamus, and brainstem [6].

Clinically, it can present in early adulthood with varied presentations like migraine with aura, encephalopathy, neuropsychiatric symptoms, ischemic strokes, cognitive impairment, and seizures [7]. The presentation of migraine with visual and sensory aura is seen in almost half of affected patients; if accompanied by motor symptoms, it can mimic an ischemic stroke [8]. Around 10% of affected people manifest acute reversible encephalopathy or CADASIL coma, which can present as hallucinations, seizures, and focal neurological deficits [9]. Ischemic stroke is present in 85% of individuals, and lacunar strokes are classic presentations [10]. At a later age, around 75% develop dementia, and cognitive decline usually follows stepwise deterioration [2]. Around 25% to 30% suffer from neuropsychiatric symptoms like adjustment disorder, depression, bipolar disorder, delusions, and hallucinations, which are also commonly noted [11]. Rare manifestations include spinal cord infarction and intracerebral hemorrhage. A positive family history and presentation at a very young age should raise suspicion for CADASIL disease. MRI is the most sensitive diagnostic test, usually showing subcortical or brainstem-circumscribed lesions on T1- and T2-weighted images [12].

In suspected cases, diagnosis is confirmed by identifying pathogenic variants of the NOTCH3 gene. Non-definitive genetic results should undergo skin biopsy to identify granular osmiophilic material in the vascular layer through electron microscopy or look for the extracellular domain of NOTCH3 receptors on the vascular media of arteries [13].

There is no definitive management for TIA or acute stroke in CADASIL, and the safety and efficacy of thrombolytics are largely unknown [14]. Current recommendations lean more towards lifestyle modification and aggressive control of blood pressure, diabetes, antiplatelet, and statin therapy. It is important to note that hypotension should be avoided perioperatively, and mean arterial pressure should be maintained at >60 mmHg and end-tidal carbon dioxide at around 40 mmHg. Conventional catheter angiography is usually avoided, as it is associated with prolonged neurological symptoms [15].

A few observational studies on the clinical prognosis of CADASIL noted that individual progression varied substantially, leading to stroke and dementia, causing moderate to severe disability and death [16-18]. Independent factors leading to poor outcomes were noted to be gait disturbances, smoking history, more than three lacunar strokes, and cerebral atrophy on imaging. The mean age of death was noted to be around 65 in male patients and 71 in female patients [2].

## Conclusions

Genetic causes of stroke should be considered in depth in patients with a strong family history of cerebrovascular accidents, even when an incidental finding like a PFO is present as a red herring. CADASIL is a genetic disease that leads to acute stroke and dementia at a very young age and can often go undiagnosed in clinical practice. Screening family members of CADASIL disease can help prevent recurrent stroke and dementia. The safety and efficacy of thrombolysis are largely unknown and should be used with caution in this population. Recent advancements in molecular genetics and early recognition of this condition with genetic testing might help risk stratification and prevent adverse cerebrovascular outcomes.
